# High pheromone diversity in the male cheek gland of the red-spotted newt *Notophthalmus viridescens* (Salamandridae)

**DOI:** 10.1186/s12862-015-0333-1

**Published:** 2015-03-25

**Authors:** Sunita Janssenswillen, Bert Willaert, Dag Treer, Wim Vandebergh, Franky Bossuyt, Ines Van Bocxlaer

**Affiliations:** Biology Department, Amphibian Evolution Lab, Vrije Universiteit Brussel (VUB), Pleinlaan 2, B-1050, Brussels, Belgium

**Keywords:** Pheromone, Urodela, Salamandridae, sodefrin precursor-like factor (SPF)

## Abstract

**Background:**

Male salamanders (Urodela) often make use of pheromones that are produced in sexually dimorphic glands to persuade the female into courtship and mating. The mental gland of lungless salamanders (Plethodontidae) and dorsal cloacal glands (or abdominal glands) of newts (Salamandridae) have been particularly well studied in that respect. In both families, sodefrin precursor-like factor (SPF) proteins have been identified as major components of the courtship pheromone system. However, similar to plethodontids, some newts also make use of subtle head glands during courtship, but few pheromones have been characterized from such structures. Males of red-spotted newts (*Notophthalmus viridescens*, Salamandridae) have both cloacal and cheek (genial) glands, and are known to apply secretions to the female’s nose by both tail-fanning and cheek-rubbing. Here we combined transcriptomic and phylogenetic analyses to investigate the presence, diversity and evolution of SPF proteins in the cloacal and cheek glands of this species.

**Results:**

Our analyses indicate that the cheek glands of male *N. viridescens* produce a similar amount and diversity of SPF isoforms as the cloacal glands in this species. Expression in other tissues was much lower, suggesting that both male-specific courtship glands secrete SPF pheromones during courtship. Our phylogenetic analyses show that *N. viridescens* expresses a combination of isoforms that stem from four highly diverged evolutionary lineages of SPF variants, that together form a basis for the broad diversity of SPF precursors in the breeding glands.

**Conclusions:**

The similar SPF expression of cheek and cloacal glands suggests that this protein family is used for pheromone signalling through cheek rubbing in the red-spotted newt. Since several male salamandrids in other genera have comparable head glands, SPF application via other glands than the cloacal glands may be more widespread than currently appreciated in salamandrids.

**Electronic supplementary material:**

The online version of this article (doi:10.1186/s12862-015-0333-1) contains supplementary material, which is available to authorized users.

## Background

Salamanders (Urodela) are known to have a wide variety of courtship dances that males use to persuade females into mating. These displays often go together with the admission of pheromones from sexual dimorphic glands that develop during the breeding season [1]. For example, in many terrestrial reproducing lungless salamanders (Plethodontidae), the mental gland hypertrophies during the mating season [2]. The secretions of this gland are either delivered through olfactory stimulation by slapping the females’ nostrils (e.g. some *Plethodon* species), or transdermally by scratching the females’ dorsum (e.g. *Desmognathus* species) [1]. In aquatically reproducing newts (Pleurodelinae, Salamandridae), males of several species develop sexually dimorphic glands in their cloaca, known as the dorsal cloacal glands^a^, which in some species extent into the pleuroperitoneal coeloem (also referred to as abdominal cavity). These glands are highly derived skin glands that are covering the caudal portion of the cloaca [3]. During courtship, newts open their cloaca to secrete pheromones, and some species additionally tail-fan towards the female for an optimal delivery [4]. In both plethodontids and salamandrids, proteins of the sodefrin precursor-like factor (SPF) system have been identified as pheromones in the male sexually dimorphic glands. In the plethodontid *Desmognathus ocoee*, behavioural experiments have shown that a fraction containing SPF proteins was able to reduce the duration of courtship [5]. In newts, it was shown that a single SPF-isoform was able to induce female following behaviour, which is one of the necessary prerequisites for successful insemination [6,7]. Whatever the exact manifestation, it seems that SPF is a pheromone system that has been able to influence female receptivity in a wide range of salamanders, probably since early urodelan evolution [7].

At first sight, specific glands and associated courtship strategies seem to be tied to particular evolutionary lineages. Indeed, studies in terrestrial plethodontids have mainly focussed on pheromone delivery using the mental gland [5,8-10], while studies in aquatically reproducing salamandrids (newts) have concentrated on pheromones that are produced by the male dorsal glands [7,11-13]. However, although tail-fanning of pheromones from the cloaca is the most obvious courtship strategy in newts, males of some species also have subtle head glands which they rub against the female’s nostrils during courtship. Although such behaviour can be found in a wide variety of newts (e.g., *Lissotriton boscai*, *Cynops pyrrhoghaster*, *Notophthalmus viridescens, Taricha granulosa*) [14-16], few pheromones have been characterized from male head glands in salamandrids.

**Figure 1:**
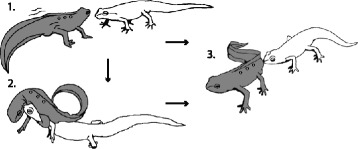
**Courtship display in** ***N. viridescens*** **.** Males are coloured in grey, females in white. (1) The male approaches the female and, if she is responsive, performs a hula-dance in front of her. (2) If the female is not responsive, the male performs an amplectic embrace using his hindlimbs, whereby he first rubs or presses his cheek against the female’s snout and subsequently starts tail waving. These behaviours are often further interchanged during courtship, indicating alternating utilization of cloacal and cheek glands. (3) A successful courtship ends with the male releasing the female from amplexus and dropping a sperm package, which the female picks up with her cloaca. See [15] for a more detailed description. Figure modified from [15].

The red-spotted newt (*Notophthalmus viridescens*) is a common salamandrid of North America for which various behavioural studies have indicated the use of chemical intra-specific communication [17-20]. Males of this species possess acinar cheek glands of which the epithelial cells enlarge during the breeding season [17]. These so-called genial glands (Latin root: gena- meaning cheek or chin [21], henceforth referred to as cheek glands) are considered to be autapomorphic for the genus *Notophthalmus*, and are present in both sexes in *N. viridescens,* although much less developed in females [17,21]. Males of this species display an amplectic embrace during which they rub their cheeks against the females’ snout, while additionally tail-waving towards her (Figure 1) [15,22]. The use of two distinct glands is likely to reflect the male’s dual strategy of pheromone delivery to persuade the female into courtship. However, while the cloacal glands of this species are known to express a wide SPF diversity [13], few precursors have been identified from the cheek glands [23] and knowledge on their diversity remains limited.

Here we combined transcriptomics and molecular phylogenetics to study the evolutionary diversity of SPF precursors in the cheek glands of the red-spotted newt. We screened and compared presence and abundance of SPF transcripts of both males and females, and compared this to the cloacal region, skin, gonads and liver. Finally, we constructed a phylogenetic tree to situate the diversity of *Notophthalmus viridescens* SPF transcripts within known salamander SPF-precursors.

## Results and discussion

### Male cheek glands express high amounts of SPF

**Figure 2:**
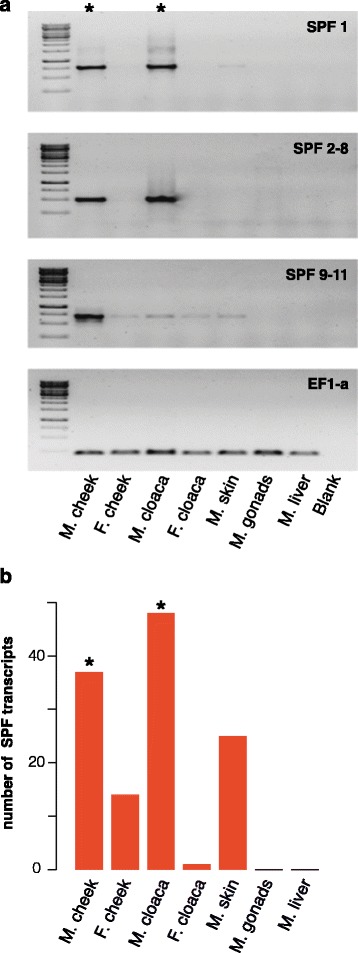
**Expression of Sodefrin Precursor Factor.** Asterisks show the male breeding glands. **(a)** PCR-Amplification of SPF precursors in the male cheek and cloaca, female cheek and cloaca, and male skin, gonads and liver, using 11 different primer combinations. The primer combinations are cross-referenced in Additional file 1. For combinations 2–8 and 9–11, comparable results were obtained and only one representative gel is shown. The elongation factor 1-alpha gene (EF1-a) was used as control. **(b)** Number of recovered SPF transcripts in breeding glands and other tissues of *Notophthalmus viridescens*.

Although SPF can be expressed in various tissues [24,25], pheromone-producing glands in salamanders generally express a substantially higher SPF precursor diversity than tissues that do not have such a role [13]. To predict a possible pheromone function for the male cheek gland, we first explored the diversity of SPF precursors in this gland, and compared it to six other tissues from sexually active animals. To have a representative screening, we used eleven primer combinations that together amplify a wide range of precursors (Additional file 1). Each of these primer combinations amplified SPF precursors from cDNA of the male cheek gland in a concentration that is comparable with, or higher than that of the male cloacal glands (Figure 2a). In contrast, only light or no gel electrophoresis bands were observed for PCR products of cDNA that was generated from equal RNA starting concentrations of other tissues (Figure 2a).

To further investigate SPF transcript diversity, we sequenced 16 clones per successfully amplified PCR product. To correct for potential PCR errors, we grouped the obtained sequences in contigs of 99% identity. This resulted in 108 unique transcripts for this species (GenBank numbers KP118895-KP118955, KM463868-KM463884, KM463886-KM463889, KM463891-KM463916) (Additional file 2), of which 15 are expressed in multiple tissues (Additional file 3). Comparative alignment of the SPF precursors (not shown) indicated that exon skipping and alternative splicing add to the observed transcript diversity, a phenomenon that is also seen in the mental gland of plethodontids and the dorsal glands of salamandrids [9,13]. We recovered 37 SPF transcripts from the male cheek glands, a diversity that is comparable to SPF expression in the cloacal glands of males (48 transcripts), but higher than the SPF diversity in other tissues investigated (Figure 2b). Together, these results indicate that SPF expression is high in both male breeding glands and suggest that males, in addition to pheromone transmission by tail-fanning, can transfer SPF protein pheromones to the female by rubbing their cheek gland directly onto her nostrils.

Because RACE-PCR can amplify products that are present in low concentrations, our approach cannot estimate the expression level of individual SPF precursors, and the relative contribution of each precursor in the cheek and cloacal glands therefore remains unknown. If SPF expression in both glands would turn out to be similar, the dual gland use with different delivery modes would not only transfer a higher quantity of SPF to the female than a single gland, but would also result in a more efficient transmission at different stages in the courtship process. Alternatively, it is also possible that different repertoires or significantly different ratios of SPF proteins are used, and that they induce different courtship responses in females. Indeed, the male initially starts cheek rubbing before he starts tail fanning, and subsequently alternates these behaviours [15]. A behavioural study with pheromone extracts from the cloacal and cheek glands of *Notophthalmus viridescens* indicated that females showed a significantly higher preference (as measured by both the amount of responsive animals and the latency of initial olfactory response) for cloacal secretions than for cheek secretions [22]. Conversely, pre-exposure to male cheek secretions accelerated initial female responses, while pre-exposure to male cloacal secretions did not show this effect, suggesting that both secretions contain a different chemical content [22]. Yet, these studies did not link their observations to SPF, and further expression analyses and behavioural testing will be necessary to unravel the exact contribution of SPF pheromones in the two glands during male courtship.

Our analyses also detected SPF expression in the skin, but to a lesser extent than in the male breeding glands (Figure 2a). Since dorsal cloacal glands are highly derived skin glands [3], continuous expression of SPF throughout the skin may have provided a pre-existing basis for favouring areas of elevated expression in glands that evolved in association with new courtship strategies. Possibly for the same reason, we also found SPF in a reduced quantity in the female cheek glands of *N. viridescens*. These glands have a similar structure and secretory activity as that of males, but there is no known behaviour that suggests the use of female pheromones during courtship [17,21].

### Phylogenetic analyses reveal highly divergent SPF precursors in *N. viridescens*

**Figure 3:**
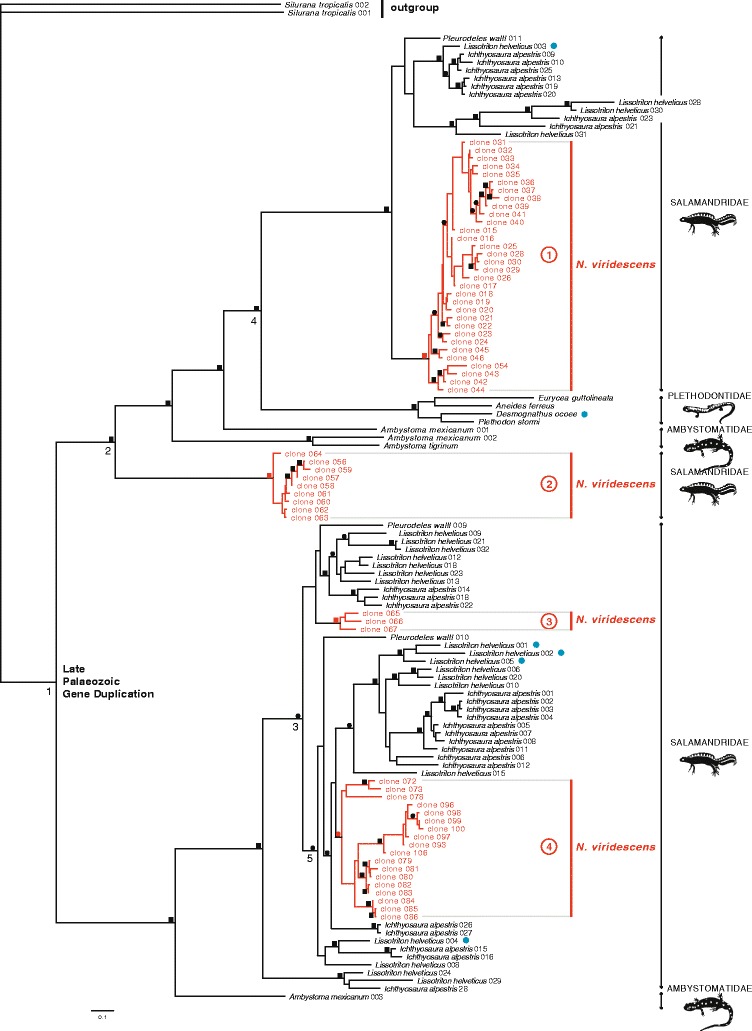
**Bayesian consensus phylogram for SPF protein diversification** ***.*** Precursor numbers are cross-referenced in Additional files 2 and 3. Squares on the branches indicate Bayesian posterior probabilities (BPP) equal to or higher than 0.95 in combination with ML bootstrap support equal to or higher than 70. Circles indicate support for BPP alone. The support for four clades of *N. viridescens* precursor sequences (clades 1 to 4) is indicated in red. Nodes 1 to 3 indicate gene duplications that mark the divergence of these SPF clades. Node 4 represents the speciation event Salamandridae-Plethodontidae, and node 5 shows the onset of newt diversification. Blue dots show SPF protein sequences that have been identified as courtship pheromones [5,7].

Although some urodelan families reproduce by external fertilization, most extant salamanders have internal fertilization, in which the female picks up the male spermatophore with her cloaca without copulation. The use of SPF has so far been demonstrated in two salamander families (Plethodontidae and Salamandridae) [5,7] that represent the basal divergence in the clade of internally fertilizing salamanders, at about 176.0 million years ago (mya) (median value from TimeTree version 12 November 2014) [26], and these courtship pheromones are therefore likely present in many species of this group [7]. However, the genes that produce the SPF protein pheromones started to diverge already in the Late Palaeozoic, well before the diversification of the internally fertilizing salamanders [7]. To investigate the evolutionary diversity of SPF precursors in *N. viridescens* in this context, we combined the mature coding sequence of a representative set of 62 sequences of this species with precursor sequences available on GenBank for salamandrids (*Lissotriton helveticus*, *Ichthyosaura alpestris* and *Pleurodeles waltl*) [7,13], ambystomatids [27,28] and plethodontids [9] in phylogenetic analyses (see Additional file 4). Two Phospholipase A2 inhibitors (PLI) frog sequences were chosen as outgroup [13,29]. Alignment resulted in a data matrix of 237 characters for 123 terminals. Maximum likelihood analyses were done under the JTT + G + I model, as assigned by ProtTest, and yielded eight trees of equal likelihood (−Ln *L* = 15631.10; four categories; shape parameter = 1.73358; assumed proportion of invariable sites = 0.0251882). The likelihood trees are consistent with supported nodes in the Bayesian consensus phylogram. All trees show that *N. viridescens* SPF precursors form four well-supported clades (Figure 3, indicated in red), differing only in phylogenetic placement of individual clones within these clades. Speciation-duplication analyses in Notung [30] identified each of the nodes that represent a divergence between *N. viridescens* SPF clades (Figure 3, nodes 1 to 3) as gene duplication events. The earliest split (Figure 3, node 1) corresponds to a duplication that happened early in urodelan evolution, and was estimated a Late Palaeozic event [7]. Two other duplications (Figure 3, nodes 2 and 3) predate the origin of the internally fertilizing clade (represented by the speciation event Salamandridae-Plethodontidae, Figure 3, node 4) and the onset of newt diversification (represented by a clade containing a *Pleurodeles waltl* sequence together with other salamandrid gene sequences, Figure 3, node 5), respectively. As a consequence, our tree indicates that *N. viridescens* expresses a combination of isoforms that stem from four highly diverged evolutionary lineages of SPF variants.

A recent study on SPF pheromones identified two conserved cysteine patterns in protein sequences of the palmate newt *Lissotriton helveticus* (Additional file 5), and showed that these SPF forms diverged early in urodelan evolution [7] (the earliest split corresponds to node 1 in our Figure 3). Furthermore, analyses of SPF in courtship water indicated that representatives of each of these proteins are secreted side-by-side by tail-fanning palmate newts [7] (Figure 3, indicated with blue dots). Our phylogenetic analyses here show that three of our *N. viridescens* precursor clades are sister to a clade that contains known palmate newt sequences. However, we also find an additional clade of ten SPF precursors (Figure 3, clade 2) for which no other urodelan representatives have been described so far. These transcripts possess a high signal peptide similarity (14 to 18 identical residues on 20 amino acids) with SPF sequences of the *N. viridescens* clade 1 precursors (Figure 3), but the mature protein is very different, and has a distinct cysteine pattern (Additional file 5). Our phylogenetic analyses suggest that these SPF precursors originated from a gene duplication that happened early in SPF evolution and that *N. viridescens* males thus express an array of anciently duplicated SPF genes. Further comparison of the levels of expression of the precursors in each of these clades will be necessary to understand their relative contribution in male courtship. Furthermore, the study of the repertoire, expression level and phylogenetic placement of SPF proteins in externally fertilizing salamanders will provide valuable information on pheromone use throughout salamander evolution.

## Conclusion

Although not every precursor necessarily codes for a functional pheromone, the wide SPF diversity in the male cheek gland of *N. viridescens* suggests that this family of proteins serves a pheromone function during cheek rubbing in this species. Our phylogenetic analyses show that *N. viridescens* expresses a combination of isoforms that stem from four highly diverged evolutionary lineages of SPF variants, that together form the basis for a broad diversity of SPF precursors in the breeding glands. SPF pheromone application via other glands than the cloacal glands may be more widespread in Salamandridae than currently assumed.

## Methods

### Ethics statement

A male and female *Notophthalmus viridescens* were purchased from a local pet shop (Squama, Herent, Belgium). The animals were first anesthetized by immersion in 0.5 g/L buffered MS-222 (Sigma-Aldrich) and then euthanized by decapitation and pithing of the brain and spinal canal. This procedure does not violate any European convention (European Convention for the protection of Vertebrate animals used for experimental and other scientific purposes; CETS #123), Belgian law (Art. 2.6 of the Belgian Law of May 4th 1995), or institutional regulation, and thus requires no approval by the Ethical Committee for Animal Research of the Vrije Universiteit Brussel.

### Species and tissue sampling

The animals were housed in a tank (H:30 cm, W:30 cm, L:50 cm) filled with water (initial water depth = 15 cm). Aquatic plants and several pieces of wood were provided as hiding places. Animals were brought into courtship mood by manipulating environmental parameters: genders were kept separately in a cold room of 6°C with an artificial day/night rhythm of 8/16 hours, and were fed bloodworms once a week. After 4 weeks, they were moved to a 12°C room with a day/night rhythm of 9/15 hours. Over a period of 3 weeks, the temperature was elevated to 18°C, the daytime was lengthened to 12 hrs, and the water depth was increased with 10 cm. Every other day, the animals were thoroughly fed bloodworms, maggots and small earthworms *ad libitum*. During these 3 weeks, the male’s breeding glands became visible and the female’s belly got swollen. Genders were brought together for mating, and the animals were subsequently anesthetized and euthanized (see ethics statement).

The following tissues were sampled in 1 ml of RNA-later (Life Technologies): cloaca, cheek glands, dorsal skin, gonads and liver of the male, and cloaca and cheek glands of the female. Distinguishing between the different types of glands in the cloaca of *N. viridescens* was unfeasible and we therefore sampled the entire cloaca (which also contains other glands), and refer to “cloacal glands” throughout the text. However, since the enlarged portion of the dorsal cloacal gland in *L. helveticus* is known to express a high diversity of SPF [7], it is likely that the SPF transcripts found in this study are mainly expressed in the dorsal cloacal glands.

### Amplification, cloning and sequencing of salamander SPF precursors

For each tissue, 1 μg total RNA (A260/A280 ratio’s between 1,7 and 1,8) was isolated with Tri Reagent T9424 (Sigma Aldrich). First strand cDNA was generated with the SMARTer RACE cDNA Amplification Kit (Clontech). The cDNA was used as template in either conventional PCRs or RACE-PCRs, both with the Advantage High Fidelity 2 PCR kit (Roche). Conventional PCR were performed with a degenerate primer pair SPF1 (SPF1for-SPF1rev) (Additional file 1) obtained from [23]*.* To compare SPF expression across tissues, the housekeeping gene Elongation Factor -1α (EF-1α) was screened in parallel (Additional file 1) [31]. PCR contents were mixed (15 μL PCR-grade water; 2,5 μL 5× HF 2PCR buffer; 1 μL DMSO; 2,5 μL 50× HF 2 dNTP mix; 1 μL Forward primer (10 μM); 1 μL Reverse primer (10 μM); 1 μL cDNA (9 ng/μL); 1 μL 50× Advantage HF 2 polymerase mix), and the PCR reaction was executed using the cycle program 94°C-3 min/4 × (94°C-45 sec/53°C-45 sec/72°C-90 sec)/30 × (94°C-30 sec, 53°C-30 sec/72°C-1 min)/72°C-7 min. For RACE-PCR, seven RACE-primers were used from [13] that were designed to maximise the phylogenetic and structural diversity of obtained SPF transcripts (primers SPF2-8) (Additional file 1). For RACE-PCR, contents were mixed (13,5 μL PCR-grade water; 2,5 μL 5× HF 2PCR buffer; 1 μL DMSO; 2,5 μL 50× HF 2 dNTP mix; 2,5 μL Universal RACE Primer Mix (UPM; 2 μM UPM1 + 0,4 μM UPM2); 1 μL SPF primer (10 μM); 1 μL cDNA (9 ng/μL); 1 μL 50× Advantage HF 2 polymerase mix) and a PCR was executed in the cycle reaction 5× (94°C-30 sec/72°C-3 min), 5 × (94°C-30 sec/70°C-30 sec/72°C-3 min), 25 × (94°C-30 sec/68°C-30 sec/72°C-3 min). Because we obtained an SPF-precursor with a cysteine pattern that strongly deviates from the known precursors (Figure 3, clade 2) (Additional file 5), we additionally designed RACE-primer SPF9 (Additional file 1) (PCR contents and reaction identical to previous RACE-PCRs), and two primer pairs (SPF10for-SPF10rev and SPF11for-SPF11rev) (Additional file 1) for a conventional PCR-reaction (PCR contents identical to previous conventional PCRs). PCR reactions with primer pairs SPF10 and SPF11 were executed using the cycle program: 94°C-3 min/4 × (94°C-45 sec/60°C-45 sec/72°C-90 sec)/30 × (94°C-30 sec, 60°C-30 sec/72°C-1 min)/72°C-7 min. After checking on a 1% electrophorese gel, PCR products were purified with the QiaQuick PCR purification kit (Qiagen) and 2 μL was used for cloning into a pGEM®-T Easy vector (PROMEGA). 2 μL of the ligation products were inserted into 50 μL TOP10 chemically competent cells (Invitrogen) that were grown overnight on LB-agar (25 mL)/Ampicillin (50 μL/mL)/X-Gal (40 μL 20 mg/mL) plates.

For each of the amplified PCR-products, we sequenced 16 clones using a Big Dye Terminator Cycle Sequencing Kit v3.1 (Applied Biosystems, PE; identical protocol as in manual) on a GeneScan 3100 automated sequencer (Applied BioSystems). Electropherograms were read using the CodonCode Aligner 3.7.1.1 software package (CodonCode Corporation). For the male cloacal gland, 12 clones of PCR products with primer pairs SPF1-8, were sequenced in a parallel study [13] and are also included here. To obtain 16 clones per primer pair, as for the other tissues, four additional cloaca clones were sequenced for this study. Nucleotide sequences of the coding region were compiled into contigs using a 99% similarity threshold after quality trimming to eliminate differences due to PCR error.

### Phylogenetic analyses

To visualize the diversity of *N. viridescens* precursors, we created a sequence alignment of *N. viridescens* SPF transcripts using the local-alignment algorithm E-INS-I with automatic parameters implemented in Mafft v7 [32] and constructed an Unweighted Pair-Group Method with Arithmethic Averaging (UPGMA) tree in PAUP* [33], (Additional file 3). To infer phylogenetic relationships of the precursors with respect to other salamanders, we selected 62 highly diverse *Notophthalmus* sequences based on our UPGMA tree. Transcript sequences were translated into the corresponding SPF precursor protein sequences with the ExPASy Translate tool [34]. We constructed a dataset including SPF protein precursor sequences of other Salamandridae, Ambystomatidae, and Plethodontidae [7,9,27,28]. Two PLI sequences of the frog *Silurana tropicalis* were added as outgroup [13] (Additional file 4). Amino acid sequences were originally aligned using the local-alignment algorithm E-INS-I with standard automatic parameters implemented in Mafft v7 [32]. ProtTest 2.4 was used to select the best fitting model of amino acid (AA) replacement for this data set according to the Akaike Information Criterion [35]. Phylogenetic relationships were estimated under maximum likelihood (ML) with PAUP* [33] under a JTT + G + I model (as assigned by ProtTest), and in a Bayesian framework using MrBayes 3.2.2 [36]. Bayesian analyses were conducted using a mixed prior for the AA substitution model, gamma correction for among-site rate heterogeneity and an estimated proportion of invariable sites. Two runs of four Markov chain Monte Carlo (MCMC) chains each were executed in parallel for 10,000,000 generations, with a sampling interval of 1,000 generations. Convergence of the parallel runs was confirmed by split frequency standard deviations (<0.01) and potential scale reduction factors (approximating 1.0) for all model parameters, as reported by MrBayes. Adequate posterior sampling was verified using Tracer 1.5 [37], by checking if the runs had reached effective sampling sizes >200 for all model parameters. A Bayesian consensus phylogram and Bayesian posterior probabilities (BPP) were inferred from the last 5000 sampled trees of both runs. Clade support under ML was assessed by 1000 replicates of rapid bootstrapping using RAXML 7.0.4 [38] on the CIPRES server [39]. Speciation-duplication analyses were done using Notung v2.6 [30].

## Endnotes

^a^Newt sexual dimorphic cloacal glands from which pheromones are released are both known as abdominal glands and dorsal cloacal glands [2]. The name abdominal gland comes from the fact that, in some newts, the dorsal cloacal gland is extended up to pleuroperitoneal coelom i.e. the so-called abdominal cavity of newts [2]. However, *N. viridescens* and other male newt species lack this enlarged portion of the dorsal cloacal glands and only contain glands located in the dorsal or dorso-lateral part of the cloaca [2]. For this reason and the fact that Urodela lack a true abdominal cavity, the term “dorsal cloacal glands” has been preferred above “abdominal glands” [2]. The dorsal glands of salamandrids are homologous with the ventral glands of plethodontids, but the latter are highly reduced in comparison to the salamandrid dorsal glands [2].

## Additional files

Additional file 1:
**Primer combinations for SPF screening in** ***N. viridescens*** **tissues.**
Additional file 2:
**Accession numbers of the individual SPF precursor numbers in Figure** 3 **and Additional file** 3 **.**
Additional file 3:
**UPGMA tree showing the diversity of SPF transcripts found in** ***N. viridescens.*** The four groups that were recovered as well-supported clades in our phylogenetic analyses are indicated. Precursor numbers are cross-referenced in Figure 3. Accession numbers matching the 108 precursor numbers are listed in Additional file 2. Additional file 4:
**Accession numbers and tissue origin of SPF precursor sequences of other amphibian species used in the phylogenetic analyses.**
Additional file 5:
**Conserved cysteine patterns per SPF clade in** ***N. viridescens.***

## References

[1] Houck LD, Arnold S, Sever D, Sever DM (2003). Courtship and mating behavior. Reproductive biology and phylogeny of Urodela.

[2] Sever DM, Sever DM (2003). Courtship and mating glands. Reproductive biology and phylogeny of Urodela.

[3] Sever DM (1992). Comparative anatomy and phylogeny of the cloacae of salamanders (Amphibia: Caudata). IV. Salamandridae. Anat Rec.

[4] Halliday TR, Slater PJB, Rosenblatt JS, Beer C (1990). The evolution of courtship behavior in newts and salamanders. Advances in the study of behavior.

[5] Houck LD, Watts RA, Mead LM, Palmer CA, Arnold SJ, Feldhoff PW, Hurst JL, Beynon RJ, Roberts SC, Wyatt TD (2008). A candidate vertebrate pheromone, SPF, increases female receptivity in a salamander. Chemical signals in vertebrates 11.

[6] Treer D, Van Bocxlaer I, Matthijs S, Du Four D, Janssenswillen S, Willaert B (2013). Love is blind: indiscriminate female mating responses to male courtship pheromones in newts (Salamandridae). PLoS One.

[7] Van Bocxlaer I, Treer D, Maex M, Vandebergh W, Janssenswillen S, Stegen G (2015). Side-by-side secretion of late Palaeozoic diverged courtship pheromones in an aquatic salamander. P Roy Soc B-Biol Sci.

[8] Rollmann SM, Houck L, Feldhoff RC (1999). Proteinaceous pheromone affecting female receptivity in a terrestrial salamander. Science.

[9] Palmer CA, Watts RA, Houck L, Picard AL, Arnold SJ (2007). Evolutionary replacement of components in a salamander pheromone signalling complex: more evidence for phenotypic-molecular decoupling. Evolution.

[10] Houck L, Palmer CA, Watts RA, Arnold SJ, Feldhoff PW, Feldhoff RC (2007). A new vertebrate courtship pheromone, PMF, affects female receptivity in a terrestrial salamander. Anim Behav.

[11] Kikuyama S, Toyoda F, Ohmiya Y, Matsuda K, Tanaka S, Hayashi H (1995). Sodefrin: a female-attracting peptide pheromone in newt cloacal glands. Science.

[12] Yamamoto K, Hayashi T, Ohe Y, Hayashi H, Toyoda F, Kawahara G (2000). Silefrin, a sodefrin-like pheromone in the abdominal gland of the sword-tailed newt, *Cynops ensicauda*. FEBS Lett.

[13] Janssenswillen S, Vandebergh W, Treer D, Willaert B, Maex M, Van Bocxlaer I (2015). Origin and diversification of a salamander sex pheromone system. Mol Biol Evol.

[14] Rafinski J, Pecio A (1992). The courtship behaviour of the Bosca’s newt, *Triturus boscai* (Amphibia: Salamandridae). Folia Biol-Krakow.

[15] Verrell P (1982). The sexual behaviour of the red-spotted newt, *Notophthalmus viridescens* (Amphibia: Urodela: Salamandridae). Anim Behav.

[16] Hippe SR, Propper CR, Staub NL (2014). The presence of sexually dimorphic submandibular glands in *Taricha granulosa*, the rough-skinned newt (Salamandridae). Copeia.

[17] Pool TB, Dent JN (1977). The ultrastructure and the hormonal control of product synthesis in the hedonic glands of the red-spotted newt *Notopthalmus viridescens*. J Exp Zool.

[18] Dawley EM (1984). Identification of sex through odors by male red-spotted newts, *Notophthalmus viridescens*. Herpetologica.

[19] Verrell PA (1985). Male mate choice for large, fecund females in the red-spotted newt, *Notophthalmus viridescens*: how is size assessed?. Herpetologica.

[20] Rohr JR, Park D, Sullivan AM, McKenna M, Propper CR, Madison DM (2005). Operational sex ratio in newts: field responses and characterization of a constituent chemical cue. Behav Ecol.

[21] Sever DM, Staub NL, Norris DO, Lopez KH (2011). Hormones, sex accessory structures, and secondary sexual characteristics in amphibians. Hormones and reproduction in vertebrates. Volume 2: amphibians.

[22] Park D, Park SR (2002). Olfactory responses of male and female red-spotted newts to sex pheromones from the opposite sex. Korean J Biol Sci.

[23] Von Reis M (2007). Evolution of sodefrin precursor pheromones in salamandrid newts [Master thesis]: Oregon State University.

[24] Maki N, Martinson J, Nishimura O, Tarui H, Meller J, Tsonis PA (2010). Expression profiles during dedifferentiation in newt lens regeneration revealed by expressed sequence tags. Mol Vis.

[25] Campbell LJ, Suárez-Castillo EC, Ortiz-Zuazaga H, Knapp D, Tanaka EM, Crews CM (2011). Gene expression profile of the regeneration epithelium during axolotl limb regeneration. Dev Dynam.

[26] Hedges SB, Dudley J, Kumar S (2006). TimeTree: a public knowledge-base of divergence times among organisms. Bioinformatics.

[27] Putta S, Smith J, Walker J, Rondet M, Weisrock D, Monaghan J (2004). From biomedicine to natural history research: EST resources for ambystomatid salamanders. BMC Genomics.

[28] Sal-site: the *Ambystoma* gene and EST database. *Ambystoma mexicanum* version 4.0. (grant no. R24OD010435). www.ambystoma.org [database on the Internet]. Accessed June 2014.

[29] Hellsten U, Harland RM, Gilchrist MJ, Hendrix D, Jurka J, Kapitonov V (2010). The genome of the Western clawed frog *Xenopus tropicalis*. Science.

[30] Chen K, Durand D, Farach-Colton M (2000). NOTUNG: a program for dating gene duplications and optimizing gene family trees. J Comput Biol.

[31] Tamori Y, Iwai T, Mita K, Wakahara M (2004). Spatio-temporal expression of a DAZ-like gene in the Japanese newt *Cynops pyrrhogaster* that has no germ plasm. Dev Genes Evol.

[32] Katoh K, Standley DM (2013). MAFFT multiple sequence alignment software version 7: improvements in performance and usability. Mol Biol Evol.

[33] Swofford DL (1998). PAUP*. Phylogenetic analysis using parsimony (*and other methods). Version 4.

[34] Gasteiger E, Gattiker A, Hoogland C, Ivanyi I, Appel RD, Bairoch A (2003). ExPASy: the proteomics server for in-depth protein knowledge and analysis. Nucleic Acids Res.

[35] Abascal F, Zardoya R, Posada D (2005). ProtTest: selection of best-fit models of protein evolution. Bioinformatics.

[36] Ronquist F, Teslenko M, van der Mark P, Ayres DL, Darling A, Höhna S (2012). MrBayes 3.2: efficient Bayesian phylogenetic inference and model choice across a large model space. Syst Biol.

[37] Rambaut A, Suchard MA, Xie D, Drummond AJ (2014). Tracer v1.6.

[38] Stamatakis A, Hoover P, Rougemont J (2008). A rapid bootstrap algorithm for the RAxML Web Servers. Syst Biol.

[39] Miller MA, Pfeiffer W, Schwartz T (2010). Creating the CIPRES Science Gateway for inference of large phylogenetic trees.

