# Supplementary material for: High pheromone diversity in the male cheek gland of the red-spotted newt Notophthalmus viridescens (Salamandridae)
Source: BMC Evol Biol. 2015 Mar 25;15:54. doi: 10.1186/s12862-015-0333-1 (PMC4379952; doi:10.1186/s12862-015-0333-1)
Supplement: Additional file 1: — Primer combinations for SPF screening in N. viridescens tissues. [file 12862_2015_333_MOESM1_ESM.pdf]

Additional file 1. Primer combinations for SPF screening in *N. viridescens* tissues.

	primer combination		primer sequence	Reference
PCR	SPF 1	For	TYCTTACTCTMYTAGCACCATGAG	[18]
		Rev	TCCTCSTCACAAGAYCAGAC	[18]
	SPF10	For	CTTTGCTTTCATATCTAGAGGAGAGTCACT	this study
		Rev	AATTTCTGCTCTGGAGACATTGACAT	this study
	SPF11	For	AGACGAAGAYCAGATACTCMGGCG	this study
		Rev	AATTTCTGCTCTGGAGACATTGACAT	this study
	Housekeeping EF 1α	For	ATCGACAAGAGAACCATCGA	[23]
		Rev	GTGATCATGTTCTTGATGAA	[23]
RACE-PCR*	SPF2	For	CCTCCYGSAGACAACACNCCCAATGGA	[11]
	SPF3	Rev	TCACATWTGTATCCATTGGGNGTGTTGTC	[11]
	SPF4	Rev	CTTCACATWTGTATCCATTGGGNGTGTTGTC	[11]
	SPF5	For	AGGAAATGCWATTGWGTGYGAAG	[11]
	SPF6	For	GTAYKGACTGCTCTGGTGAG	[11]
	SPF7	For	AGCTACAGGAAANKCTATTGWSTGTG	[11]
	SPF8	For	TAGTAYKGACTGCTCTGGTGA	[11]
	SPF9	Rev	GCCGGTGCAYTCCATRGTTTC	this study
Universal	UPM1		CTAATACGACTCACTATAGGGC	n.a.
	UPM2		CTAATACGACTCACTATAGGGCAAGCAGTGG TATCAACGCAGAGT	n.a.

*used in combination with the Universal RACE primer kit (UPM1 and UPM2) included in the SMARTer RACE cDNA Kit (CLONTECH).
