# Supplementary material for: High pheromone diversity in the male cheek gland of the red-spotted newt Notophthalmus viridescens (Salamandridae)
Source: BMC Evol Biol. 2015 Mar 25;15:54. doi: 10.1186/s12862-015-0333-1 (PMC4379952; doi:10.1186/s12862-015-0333-1)
Supplement: Additional file 2: — Accession numbers of the individual SPF precursor numbers in Figure 3 and Additional file 3 . [file 12862_2015_333_MOESM2_ESM.pdf]

Additional file 2. Accession numbers of the individual SPF precursor in Figure 3 and Additional file 3.

clone	GenBank nr.	clone	GenBank nr.	clone	GenBank nr.	clone	GenBank nr.
1	KM463896	28	KP118913	55	KP118896	82	KP118951
2	KM463916	29	KP118914	56	KP118897	83	KP118952
3	KM463901	30	KP118915	57	KP118898	84	KP118950
4	KM463902	31	KP118902	58	KP118899	85	KP118954
5	KM463899	32	KP118903	59	KP118928	86	KP118955
6	KM463900	33	KP118943	60	KP118941	87	KM463875
7	KM463903	34	KP118905	61	KP118940	88	KM463888
8	KM463897	35	KP118910	62	KP118900	89	KM463876
9	KM463904	36	KP118929	63	KP118901	90	KM463889
10	KM463911	37	KP118930	64	KP118895	91	KM463884
11	KM463909	38	KP118933	65	KM463868	92	KM463892
12	KM463905	39	KP118931	66	KM463869	93	KP118918
13	KM463906	40	KP118934	67	KM463870	94	KM463893
14	KM463908	41	KP118932	68	KM463871	95	KM463895
15	KP118904	42	KP118937	69	KM463874	96	KP118917
16	KP118906	43	KP118938	70	KM463873	97	KP118924
17	KP118944	44	KP118939	71	KM463880	98	KP118922
18	KP118908	45	KP118948	72	KP118916	99	KP118923
19	KP118909	46	KP118949	73	KP118919	100	KP118925
20	KP118935	47	KM463907	74	KM463879	101	KM463878
21	KP118911	48	KM463912	75	KM463882	102	KM463883
22	KP118936	49	KM463914	76	KM463872	103	KM463886
23	KP118946	50	KM463915	77	KM463881	104	KM463894
24	KP118947	51	KM463913	78	KP118920	105	KM463891
25	KP118907	52	KM463910	79	KP118921	106	KP118926
26	KP118945	53	KM463898	80	KP118953	107	KM463877
27	KP118942	54	KP118912	81	KP118927	108	KM463887
