# Supplementary material for: High pheromone diversity in the male cheek gland of the red-spotted newt Notophthalmus viridescens (Salamandridae)
Source: BMC Evol Biol. 2015 Mar 25;15:54. doi: 10.1186/s12862-015-0333-1 (PMC4379952; doi:10.1186/s12862-015-0333-1)

Additional file 3. UPGMA tree showing the diversity of SPF transcripts found in *N. viridescens*. The four groups that are recovered as well-supported clades in our phylogenetic analyses are indicated. Precursor numbers are cross-referenced in Figure 3. Accession numbers matching the 108 precursor numbers are listed in Additional file 2.

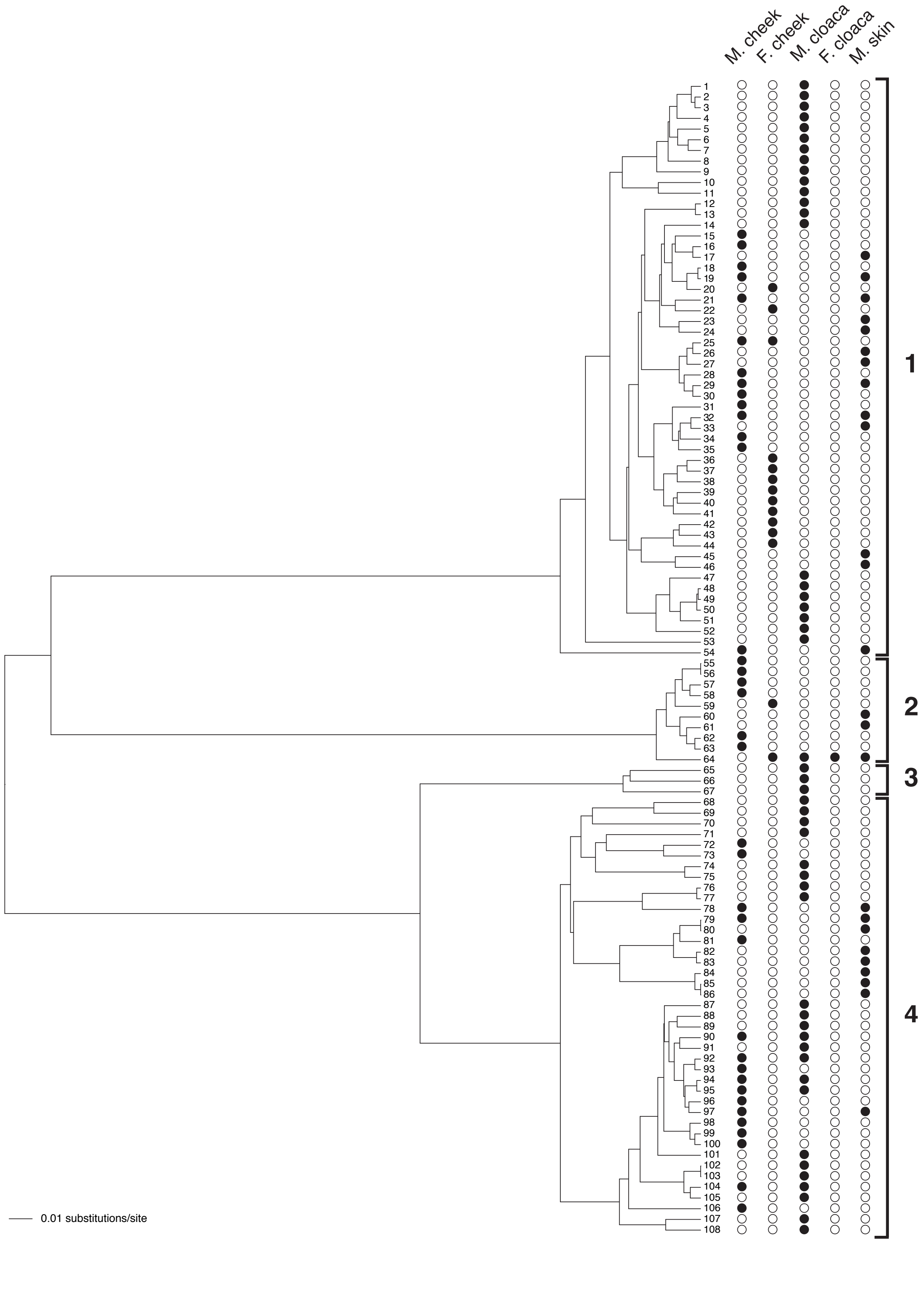

Supplement: Additional file 3: — UPGMA tree showing the diversity of SPF transcripts found in N. viridescens. The four groups that were recovered as well-supported clades in our phylogenetic analyses are indicated. Precursor numbers are cross-referenced in Figure 3. Accession numbers matching the 108 precursor numbers are listed in Additional file 2. [file 12862_2015_333_MOESM3_ESM.pdf]
