# Supplementary material for: High pheromone diversity in the male cheek gland of the red-spotted newt Notophthalmus viridescens (Salamandridae)
Source: BMC Evol Biol. 2015 Mar 25;15:54. doi: 10.1186/s12862-015-0333-1 (PMC4379952; doi:10.1186/s12862-015-0333-1)
Supplement: Additional file 4: — Accession numbers and tissue origin of SPF precursor sequences of other amphibian species used in the phylogenetic analyses. [file 12862_2015_333_MOESM4_ESM.pdf]

Additional file 4. Accession numbers and tissue origin of SPF precursor sequences of other amphibian species used in the phylogenetic analyses.

species	Accession number	tissue	database
Lissotriton helveticus 001-006	KJ402326-KJ402331	male abdominal gland	NCBI
Lissotriton helveticus 008-010	KJ402333-KJ402335	male abdominal gland	NCBI
Lissotriton helveticus 012-013	KJ402337-KJ402338	male abdominal gland	NCBI
Lissotriton helveticus 015	KJ402340	male abdominal gland	NCBI
Lissotriton helveticus 018	KJ402343	male abdominal gland	NCBI
Lissotriton helveticus 020-021	KJ402345-KJ402346	male abdominal gland	NCBI
Lissotriton helveticus 023-024	KJ402348-KJ402349	male abdominal gland	NCBI
Lissotriton helveticus 028-032	KJ402353-KJ402357	male abdominal gland	NCBI
Ichthyosaura alpestris 001-016	KP849562-KP849577	male abdominal gland	NCBI
Ichthyosaura alpestris 018-023	KP849579-KP849584	male abdominal gland	NCBI
Ichthyosaura alpestris 025-028	KP849586-KP849589	male abdominal gland	NCBI
Pleurodeles waltl 009	KM463930	male cloacal tissue	NCBI
Pleurodeles waltl 010	KM463931	male cloacal tissue	NCBI
Pleurodeles waltl 011	KM463932	male cloacal tissue	NCBI
Eurycea guttolineata	AAZ06338	male mental gland	NCBI
Aneides ferreus	AAZ06335	male mental gland	NCBI
Desmognathus ocoee	AAZ06329	male mental gland	NCBI
Plethodon stormi	DQ097067	male mental gland	NCBI
Ambystoma mexicanum 001	CN041146	larval limb tissue	NCBI
Ambystoma mexicanum 002	CN035733	larval limb tissue	NCBI
Ambystoma mexicanum 003	I1698676-GIB5VLK02G78TL	brain, blood, etc.	SGP/Sal-site
Ambystoma tigrinum	CN048649	brain tissue	NCBI
Silurana tropicalis 001	F6PQG9	n.a.	Uniprot
Silurana tropicalis 002	XP_002943341	n.a.	NCBI
