# Supplementary material for: High pheromone diversity in the male cheek gland of the red-spotted newt Notophthalmus viridescens (Salamandridae)
Source: BMC Evol Biol. 2015 Mar 25;15:54. doi: 10.1186/s12862-015-0333-1 (PMC4379952; doi:10.1186/s12862-015-0333-1)
Supplement: Additional file 5: — Conserved cysteine patterns per SPF clade in N. viridescens. [file 12862_2015_333_MOESM5_ESM.pdf]

Additional file 5. Conserved cysteine patterns per SPF clade in *N. viridescens*.

```
                         signal peptide                      mature protein
clade1     SPF032 MKALLASISILFAFISRGNAIECEVCSGQASTD-CSGELVT-CDQTVESCQTAITDMTFEGLDSMYVISKNCSDVGAK-NILYRVAAKDV--FYQQ-RVEVCQTNGCNKGPLQFPPKNTTLNG
clades3,4 SPF094 MRAILAAVVMLQALITGADCLLCEQCFVVGSSQ-CSGIFKQ-CSPDVTHCVKGMENSTLGTRVVLSAF-KDCLNPSQKAACGREFFHKDSALFFQITRT-CCDSDFCNKRDVEVPAVDNTPNG
clade2     SPF063 MKALLASVSILFAFISRGESLSCEQCMNLNGAT-CSGNQRV-CEGYATRCDNTYMEFTRDGQTTSTTF-KGCAVEQK---CTNYFLSESLGGFQFRMQKSYCEKDNCNTRDLVVPPRNNTPNG

clade1     SPF032                     VKCPTCVVDGELS CESTEVLECVGAMTNCLYFAATFRNTAAPPVQTAFRGCTNAKFAEQVPIGPPYTVQDVVT--LIV---S---KGV
clades3,4 SPF094                     YKCEDCFTTQSTGPCTATGGIQCTGEQNTCGSFSGGVARPGEVVRQISMKGCVSPDF---CDLFYPAATQ-VYNYDLL---CSPAEKL
clade2     SPF063                     VRCPKCYAENATS CQSYETMECTGLETKCMDFGGKIRKYDQILTVASMN-CVNEEP---CNQPIMYCSDGPAVEIDHFR-CSDGEKYS
```
